# Hybrid Optical Unobtrusive Blood Pressure Measurements

**DOI:** 10.3390/s17071541

**Published:** 2017-07-01

**Authors:** Guangfei Zhang, Caifeng Shan, Ihor Kirenko, Xi Long, Ronald M. Aarts

**Affiliations:** 1 Philips Research Eindhoven, 5656 AE Eindhoven, The Netherlands; guangfei.zhang.private@gmail.com (G.Z.); ihor.kirenko@philips.com (I.K.); xi.long@philips.com (X.L.); ronald.m.aarts@philips.com (R.M.A.); 2 Department of Electrical Engineering, Eindhoven University of Technology, 5612 AZ Eindhoven, The Netherlands

**Keywords:** unobtrusive BP measurement, photoplethysmography, imaging photoplethysmography, pulse transit time

## Abstract

Blood pressure (BP) is critical in diagnosing certain cardiovascular diseases such as hypertension. Some previous studies have proved that BP can be estimated by pulse transit time (PTT) calculated by a pair of photoplethysmography (PPG) signals at two body sites. Currently, contact PPG (cPPG) and imaging PPG (iPPG) are two feasible ways to obtain PPG signals. In this study, we proposed a hybrid system (called the ICPPG system) employing both methods that can be implemented on a wearable device, facilitating the measurement of BP in an inconspicuous way. The feasibility of the ICPPG system was validated on a dataset with 29 subjects. It has been proved that the ICPPG system is able to estimate PTT values. Moreover, the PTT measured by the new system shows a correlation on average with BP variations for most subjects, which could facilitate a new generation of BP measurement using wearable and mobile devices.

## 1. Introduction

Blood pressure (BP) is an indispensable vital sign to evaluate human health. Catheterization is the gold standard method to measure the BP of humans, which measures instantaneous BP by placing a strain gauge in fluid contact with blood at any arterial site (e.g., radial artery, aorta) [1]. The main drawback of catheterization is the invasiveness, which is unacceptable for healthy subjects in their daily lives. At present, there are several surrogate non-invasive methods to measure BP in clinical practice, such as auscultation [2], oscillometry [3], volume clamping [4], and tonometry [5]. However, the existing conventional BP measurement methods are either manual, cumbersome, or require a cuff, which are time-consuming and disruptive during measurements. The next generation of BP measurements should be ultra-convenient, unobtrusive, easy to implement and portable, which will benefit the ambulatory monitoring of population health in terms of prevalent diseases such as arterial hypertension, hypotension, and other cardiac dysfunction.

Pulse transit time (PTT) is the time delay for the arterial blood pressure (ABP) pulse to travel between two arterial sites. ABP pulse is acquired in an invasive way such as catheterization [6,7]. Previous research found PTT can be obtained by photoplethysmography (PPG) signals as well, which have been shown to associate with BP in an inverse fashion [8,9,10,11,12,13,14,15,16]. It is usually calculated as the temporal difference between the morphologic valleys, peak, or maxima in first derivation of PPG signals at different arterial branches. Currently, contact PPG (cPPG) and imaging PPG (iPPG) are two feasible techniques to acquire the PPG signal [17]. cPPG uses a sensor containing a simple and low-cost optical component that can detect blood volume changes in the micro-vascular bed of tissue, while iPPG employs a monochrome, RGB (red-green-blue), or IR (infrared) camera to acquire PPG signals remotely under an ambient illumination with, for example, a light-emitted-diode (LED) lamp [18,19,20,21,22,23,24,25,26,27,28,29,30].

It is known that PTT can be measured by a pair of PPG signals collected from two different body sites that either are located on a same arterial branch (i.e., wrist and finger) [31] or different arterial branches (i.e., forehead to hand) [10,32,33]. Chen et al. [10] employed two cPPG sensors to measure the PTT between the ear and the toe to estimate diastolic BP (DBP). Their estimation performance can meet the requirement of an error range in clinical condition. However, they did not provide the result in systolic BP (SBP) estimation by PTT. Jeong et al. [32] used a high-speed video camera with a sampling rate at 420 frames per second (fps) to derive the PTT between face and palm, where a negative correlation between PTT and SBP was found. In the study by Kazuya et al. [33], PTT were derived between forehead and finger using a CMOS camera with an ultra-high frame rate (at 500 fps) in a supine position. It was found that the pulse wave velocity (PWV), which is the ratio of the PTT to the distance between two arterial sites, would increase with increasing SBP. Although these camera-based methods provided promising results, they still require subjects to have a certain position or posture during measurement, which is not suitable for unobtrusive BP measurements. Moreover, using multiple cPPG sensors placed on the body is still not easily acceptable for continuous measurement due to its obtrusiveness. Using a single iPPG technique is still obtrusive, since it requires that both body sites can be captured by the camera, i.e., face and palm [32], or use multiple cameras for each site [33]. Some previous work employed a low-frame-rate camera to extract an iPPG signal for PTT calculation. For example, Balvinder et al. [19] used a camera with a 59.9 frame rate and implemented an up-sampling technique to increase the temporal resolution. Sun et al. [21] found that the negative influence of a low initial sample rate could be compensated by interpolating the signal to improve the time domain resolution and pulse rate variability (PRV) measurements. Kyal et al. [23] used cubic interpolation that is then applied to up-sample the signal to increase the number of data points for improved interval detection and to match with the sampling frequency of the recorded electrocardiography (ECG) signals.

**Figure 1:**
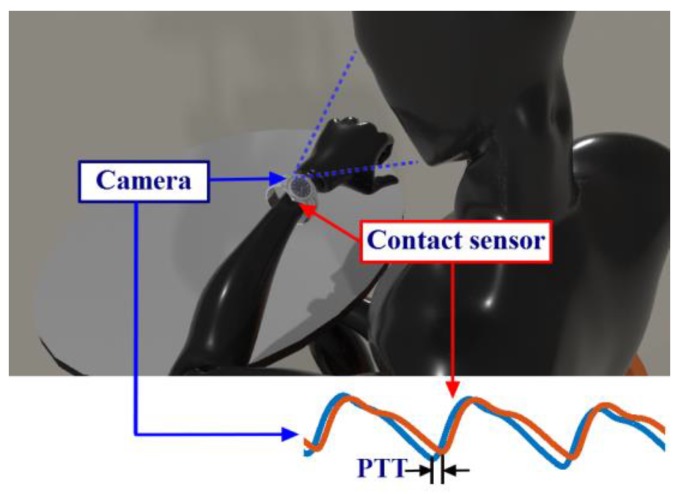
Use case of the ICPPG system (combination of contact photoplethysmography (cPPG) and imaging photoplethysmography (iPPG)). The ICPPG system can be implemented on a wristwatch with a contact photoplethysmography (PPG) sensor at the back side and a camera on the top. The pulse transit time (PTT) can be calculated by the temporal difference between the morphologic valleys of PPG signals at the wrist and the iPPG signal at the face.

In this paper, we propose a method to measure PTT by using a system combining cPPG and iPPG, called the ICPPG system. This system is able to measure the PTT between PPG signals collected at two distal arterial sites (one with cPPG and the other with iPPG). Figure 1 illustrates the conceptual scheme of the ICPPG system, where one PPG signal is acquired using the camera from a smartwatch (i.e., iPPG) and the other PPG signal is measured using cPPG sensor in contact with the skin. Along with the fast development of intelligent wearables and smartphones, the prospect of implementing our proposed ICPPG system into more digital devices is promising. It would promise unobtrusive PTT and BP measurements and is expected to be widely accepted in real-life applications.

To examine the validity of the method, we measured the PTT between face and hand, where the iPPG signal was obtained from the face and the cPPG signal at the fingertip. The results were thereafter compared with a previously validated system using two cPPG sensors placed on two sites adjacent to the sites of our method (head and wrist). Afterward, the correlation between the PTT (between two distal sites) and BP was derived.

The main contributions of this study are: (1) we proposed a novel concept for unobtrusive surrogate BP measurement by using PPG sensor and iPPG sensor corporately; (2) The feasibility of the proposed setup was investigated on the dataset with 29 subjects.

## 2. Methods and Materials

### 2.1. Methods

In this study, two pairs of PPG signals were collected by the proposed method and a conventional physiological measurement system (the gold standard), respectively. The IC-PTT (and Ref-PTT) was the PTT calculated based on the pair of PPG signal collected by the proposed method (and the gold standard measurement), and afterward, a comparison between IC-PTT and Ref-PTT was carried out. Finally, the correlation between IC-PTT (and Ref-PTT) and BP was investigated. For deriving the IC-PTT and Ref-PTT, signal processing and data analysis consisting of the following steps were implemented.

#### 2.1.1. iPPG Signal Extraction

An existing method based on spatial-intensity was applied to extract the iPPG signal, which computes the spatial average of the green channel intensity of pixels with a certain region of interest (ROI) [18,23]. In those studies, the green channel trace was used because it contains the strongest plethysmographic signal among all three (RGB) channels. In fact, the hemoglobin absorbs more light around the green wavelength compared to others. Here the cheek area was considered as ROI due to the presence of more uniformly distributed illumination on the cheek compared with the forehead. According to our previous work [34], the PTT is varied with skin areas on the face because of the different relative distances to the central artery, and the rambling distribution of the peripheral artery over the face.

**Figure 2:**
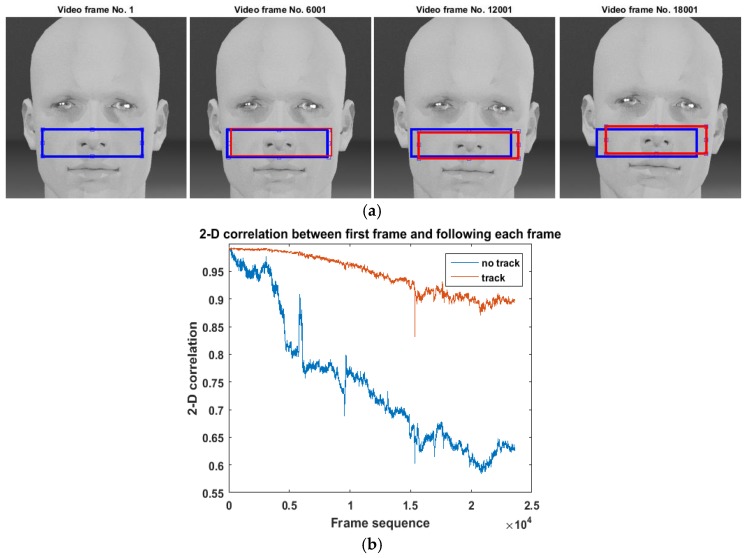
IPPG signal extraction and region of interest (ROI) selection. (**a**) An example of fixed ROI (blue block) and the tracking ROI (red blocks) with the increasing number of frames. It was found that the tracking ROI is able to adapt to the movement of the participant while fixed ROI cannot; (**b**) An example of the 2-D correlation between the skin within ROI and the original template of the skin that was selected in the first video frame vs. number of frames. It shows that the 2-D correlation of the skin within tracking ROI is higher and more stable than fixed ROI.

Conventionally, the ROI is identified by manually selecting a fixed area for each frame [10,19,32,33]. However, the head of participants could slightly move during the measurement, resulting in a shift of the skin area (see Figure 2a). This would confuse the variations in iPPG induced by physiological reactions and by skin shift. Therefore, we employed a template-based tracking ROI algorithm to adaptively track the target ROI when extracting iPPG signals in order to compensate for slight head movements. A similar image processing method was implemented in a previous study to compensate for the shifting of the subject between two frames [35]. The pre-defined ROI of the first frame was set to be a template. A two-dimensional correlation was then calculated between the template and the possible ROIs in each following frame, which is calculated by:

(1) $$C\left(k,l\right)=\overset{M-1}{\underset{m=0}{\sum }}\overset{N-1}{\underset{n=0}{\sum }}X\left(m,n\right)\bar{H}\left(m-k,n-l\right),\;\{\begin{array}{c} -\left(P-1\right)<k<M-1\\ -\left(Q-1\right)<l<N-1 \end{array}$$

*X* is a *M*-by-*N* image, which is the entire image of a frame. *H* is a *P*-by-*Q* image, which is the initially selected ROI (cheek area) in the first frame and $\bar{H}$ denotes complex conjugation. *C* is a correlation coefficient matrix with size *M* + *P* − 1 by *N* + *Q* − 1. The position of the region of the frame that matching the initially selected ROI should give the highest *C* value. Therefore the ROI showing the highest correlation to the template was identified as the target ROI in that frame, from which the iPPG data was measured. Since in this case the ROI was partially chopped from the image, the non-skin area is negligible compare to using entire image. Figure 2b compares the correlation coefficient with and without ROI tracking for an (example) sequence of frames in our experiment. It can be seen that the (adaptive) tracking ROI was able to mitigate the influence caused by slight head movements.

Note that the PPG waveforms with a low signal quality (evaluated by the metric proposed by Li et al. [36]) were excluded. As a result, 8.8 ± 4.8% (minimum: 3.5%; maximum: 22%) of Ref-PTT values were excluded, and 9.8 ± 8.9% (minimum: 3.6%; maximum: 40%) of IC-PTT values were excluded.

#### 2.1.2. PTT Calculation

**Figure 3:**
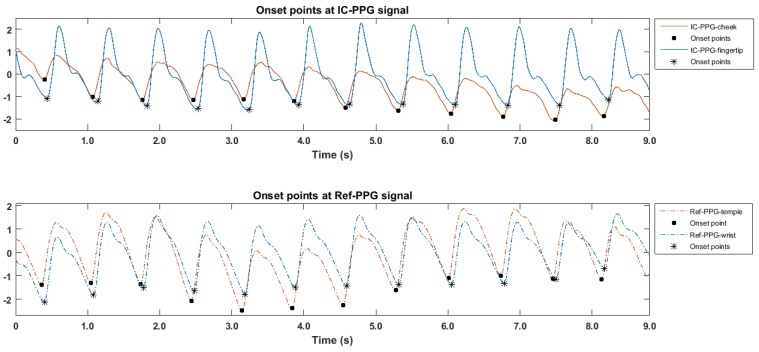
PTT calculation. The IC-PTT (combination of imagery and contact PTT) (and the Ref-PTT, a conventional physiological measurement system) calculated by the temporal difference between the onset points for each beat in the pair of IC-PPG signals (and the Ref-PPG signal).

Estimating PTT requires the localization of a characteristic point in the waveform of the PPG signal. Zong et al. proposed a morphology-based method to detect the onset of ABP pulse [37], showing a high accuracy in ABP beat detection when tested on different data sets [38,39,40,41,42]. The algorithm is in cooperation with a resample technique (to 125 fps) to enhance temporal resolution. According the documentation of the resample in MATLAB, the resampling applies an antialiasing FIR lowpass filter to the original signal and compensates for the delay introduced by the filter. By employing the algorithm of ABP beat detection, the time difference between each two onset points (see Figure 3) caused by the same heartbeat (i.e., one from the iPPG and the other from the cPPG) were derived, the PTT value (called IC-PTT) can then be obtained per heartbeat. Similarly, the Ref-PTT values can be obtained using the reference PPG system with two cPPG sensors.

The PPG signals from the finger and the wrist were drastically distorted due to the obstruction by the cuff during each BP measurement period. Therefore, the IC-PTT (and Ref-PTT) values for each BP measurement were estimated by taking an average of the IC-PTT (and Ref-PTT) values in 30-s episodes of signals before and after the corresponding BP measurement; for this, the PTT for the one-minute period around the BP measurement was calculated and given statistically as following:

${\bar{I}}_{g}\left(t\right)$ is the continuously extracted iPPG signal at the cheek. $\bar{S}\left(t\right)$ is the PPG signal collected at the finger. For each BP measurement, two corresponding episodes of both ${\bar{I}}_{g}\left(t\right)$ and $\bar{S}\left(t\right)$ were selected for PTT calculation. The episode before the BP measurement ${\bar{I}}_{g\_before}\left(t,t+T_{0}\right)$ and ${\bar{S}}_{before}\left(t,t+T_{0}\right)$, and the episode after the BP measurement ${\bar{I}}_{g\_after}\left(t,t+T_{0}\right)$ and ${\bar{S}}_{after}\left(t,t+T_{0}\right)$, where $T_{0}$ was the length of the episode (30 s in the study). A overly long episode reflects less relationship with the time point of the BP measurement, while an overly short episode is unable to provide enough beats with good quality. Therefore the length of each signal episode was defined as 30 s, which is a trade-off between relativity and quantity. The timestamp of all onset points within the episode were detected by the algorithm of ABP beat detection. The algorithm resample the input signal to 125 Hz. The same signal pre-processing can be also referred to the work by [19,21,23,30]. All onset points for each episode were detected as some timestamps such as $t_{Ippg\_onset\_before,i}$, $t_{ppg\_onset\_before,i\;}$, $t_{Ippg\_onset\_after,j}$, and $t_{ppg\_onset\_after,j}$*.* The beat-to-beat PTT for each onset point can be calculated by:

(2) $$PTT_{before,i}=t_{ppg\_onset\_before,i}-t_{Ippg_{onset_{before}},i}\;\left(i=0,1,2,\ldots ,x-1\right)$$

(3) $$PTT_{after,j}=t_{ppg\_onset\_after,j}-t_{Ippg_{onset_{after}},j}\;\left(j=0,1,2,\ldots ,y-1\right)$$

Afterwards, the PTT for each BP measurement was estimated by:

(4) $$PTT_{estimate}=\frac{\sum_{i=0}^{i=x-1}PTT_{before,i}+\sum_{j=0}^{j=y-1}PTT_{after,j})}{2+\sum_{i=0}^{i=x-1}i+\sum_{j=0}^{j=y-1}j}\;\left(i=0,1,2,\ldots ,x\right)\left(j=0,1,2,\ldots ,y\right)$$

where the *x* and *y* is the amount of onset points within the batch of signal before and after the BP measurement, respectively.

The last BP value was excluded since no PPG signals (IC-PTT and Ref-PTT) were observed after the BP measurement. The reason that averaging or taking the median of the PTT estimates over a number of beats is that it can mitigate the effects of measurement and physiologic (e.g., respiratory) artifacts, as well as erroneous beat detections [43,44].

Furthermore, the association between the estimated the IC-PTT values based on our ICPPG system and the standard BP measures (in mmHg) was assessed. In some studies, a ratio of PTT to the distance between two arterial sites was derived as PWV to estimate BP by investigating a universal model [15,33,45,46]. Since PWV and PTT are equivalent, in this study we only analyzed the association between PTT and BP variations. A Person’s correlation coefficient between BP and IC-PTT and between BP and Ref-PTT were compared. The IC-PTT, Ref-PTT, and BP values for all participants under each BP measurement are recorded as the table in appendix A.

### 2.2. Experiment and Data

**Figure 4:**
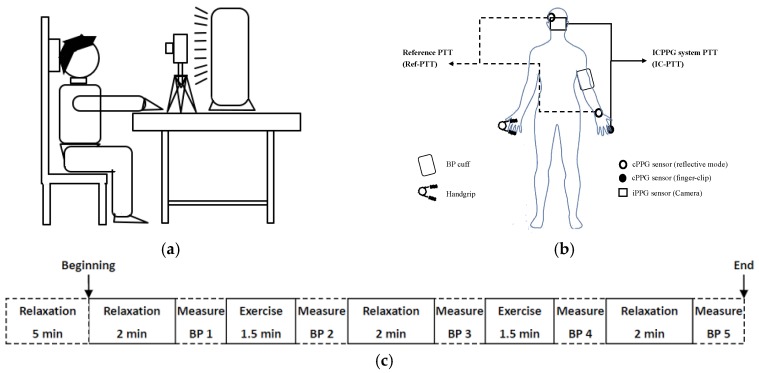
Data collection. (**a**) Setup for data collection; (**b**) Schematic overview of the data collection on the subject; (**c**) Sessions with and without exercise. Note that IC-PTT is the PTT produced by the ICPPG system and Ref-PTT is the PTT produced by the reference system.

The experiment was carried out on 29 participants (8 females). Each participant provided informed consent. None of them had been diagnosed with cardiovascular diseases. As shown in Figure 4a, the participants were asked to sit still on the chair comfortably and silently with supports on the arm, hand, back and head during the experiment. All the illumination except for the fluorescent lamp (model HF3319/01 PHILIPS) was turned off during the experiment. The fluorescent lamp was placed on a stable desk 60 cm in front of the participant. A digital camera (IDS-uEye camera model: “sensor = UI222xSE-C”) was located on the same desk 50 cm in front of the participant to capture iPPG signal from the face area. The resolution of each video frame was 300 × 300 pixels and the frame rate was fixed at 25 frame per second (fps). In addition, the sequences of frames were saved in BMP format without any preprocessing and auto-optimization from the camera. A commercial finger-clip oximeter (PULOX CMS50E) was clipped on the index fingertip to acquire the cPPG signal at the finger. The oximeter employs a contact PPG sensor with a red LED and it was synchronized with the signal from the camera. The oximeter employs a transmissive PPG sensor, thus it cannot be placed on the wrist area. The PTT values derived from the iPPG and the oximeter-based cPPG in our ICPPG system are called IC-PTT measures. In order to obtain the reference PTT (Ref-PTT) measures, a commercial physiological acquisition equipment (BIOPAC MP150) with two cPPG sensors with a green LED was used. It acquired a pair of PPG signals with a sampling frequency of 1000 Hz from the wrist and the temple of the participant. For acquiring the reference of BP, an electronic sphygmomanometer with cuff (MICROLIFE BP3AC1 with an error ±3 mmHg) was used to measure the BP including SBP and DBP at the end of each session. Figure 4b shows locations of all sensors, the cuff for BP measurement, and the tool for exercising. To prevent potential injure and fatigue due to the overloaded tension at the same arm due to the high-intensity exercise and stress from the BP cuff, we placed the BP cuff and handgrip in separate arms.

Before the experiment, each participant took a rest for five minutes. The experiment consists of five sessions with and without exercise as shown in Figure 4c, where each session started with PPG measurement during either a 2-min relaxation or a 1.5-min exercise, followed by BP measurement. The exercise session (corresponding to an increased BP) and the relaxation session (corresponding to a decreased BP) were performed alternately. For selecting the optimal type of exercise, several exercises were performed including cycling, jumping, and keep holding a handgrip. Since iPPG is less reliable with large motions, holding a handgrip was selected as the exercise to perform in this study.

## 3. Results

### 3.1. Morphologic Differences between PPG Signals

**Figure 5:**
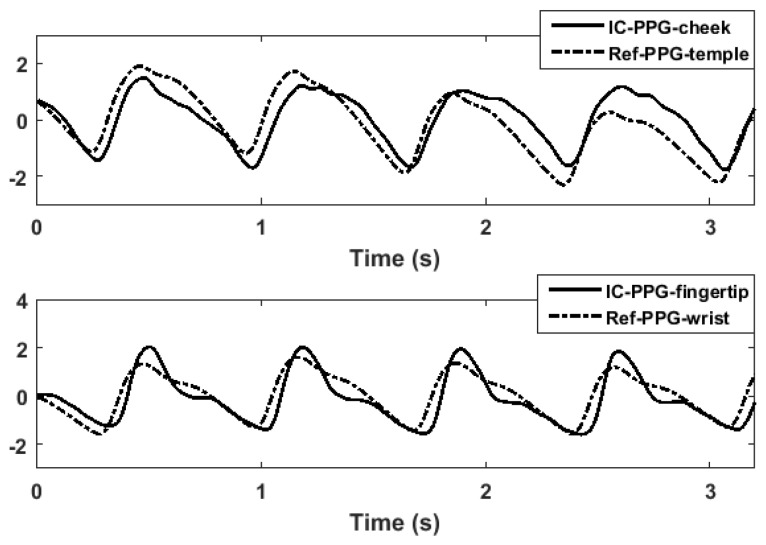
Morphologic differences between PPG signals. An example of the morphologic difference of PPG signals at different sites with different sensing modalities.

Figure 5 shows an example of a PPG signal produced by the ICPPG system and the corresponding reference signal. The morphologic difference observed in the figure might be caused by the difference of tissue where the PPG sensor was placed, or the difference in PPG sensing modalities. The sensitivity of the PPG morphologic difference and its influence on PTT measurement should be investigated in future work. From the figure, we found that the ICPPG signals and their reference signals showed the aligned periodicity. This indicates that the ICPPG system can reveal the same heartbeat as reference system does, which is in line with the previous finding that the facial color variation is related to the blood volume changes induced by heart pumping [17].

### 3.2. Consistency between IC-PTT and Its Reference

**Figure 6:**
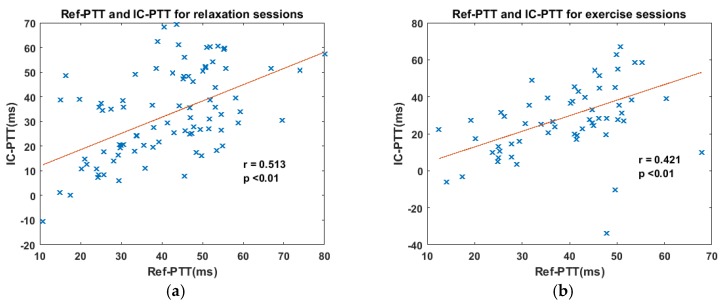
The correlation between IC-PTT and Ref-PTT for relaxation and exercise scenarios. (**a**) The correlation between IC-PTT and Ref-PTT of relaxation sessions; (**b**) The correlation between IC-PTT and Ref-PTT of exercise sessions.

It was found that IC-PTT was correlated with Ref-PTT in both the relaxation sessions (r = 0.513, *p* < 0.01) and exercise sessions (r = 0.421, *p* < 0.01), as shown in Figure 6. The correlation coefficient during exercise sessions is slightly lower than that during relaxation sessions, likely due to more motion artifacts during physical exercise. The significant correlations indicate the validity of our ICPPG system in reliably measuring PTT values, even when the subjects were carrying out physical exercises. However, the performance of the ICPPG system can be affected by artifacts such as motion.

### 3.3. Variability of IC-PTT and Its Reference

**Figure 7:**
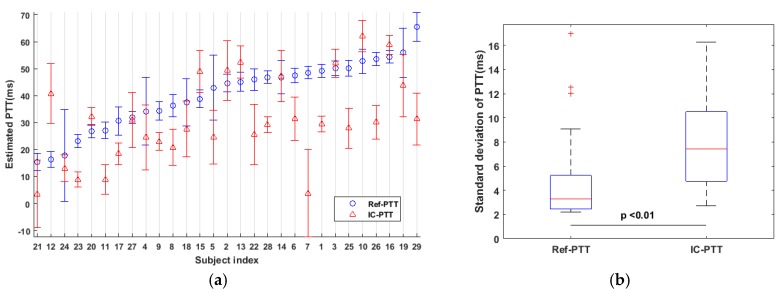
The variability of IC-PTT and Ref-PTT. (**a**) The mean value and standard deviation of IC-PTT and Ref-PTT for each subject during the experiment; (**b**) Standard deviations of IC-PTT and Ref-PTT. There is a significant difference between the variability of IC-PTT and Ref-PTT.

The mean value and standard deviation of IC-PTT and Ref-PTT values during all sessions were investigated as shown in Figure 7a. It can be observed that the PTT measurements varied across subjects, which can be explained by inter-individual variability in physiology, such as the difference in arterial elasticity between subjects. In addition, IC-PTT shows larger variability than Ref-PTT. The mean variability of IC-PTT during the whole experiment is 7.65 ± 3.59 ms. The mean variations of IC-PTT and Ref-PTT are 7.65 ± 3.59 ms and 4.77 ± 3.67 ms, respectively, as shown in Figure 7b. It appears that there is a significant difference (paired *t*-test) between the variability of IC-PTT and Ref-PTT, which could be caused by the different pressure exerted on the tissue using different sensing modalities or the larger skin area that measured by the iPPG sensor. In addition, it was experimentally found that the Ref-PTT was larger than IC-PTT, which might be explained by the difference modalities or mechanisms of PPG sensors used in ICPPG system and the reference system. The PPG sensor used in ICPPG system is a transmissive mode but the one used in the reference system is a reflective mode, meaning that IC-PTT can be measured from a deeper artery instead of the capillary where the ABP pulse consumes more time to arrive. In this study, the mean values of IC-PTT were at the range from 10 ms to 65 ms. As reported by Allen [47], the inter-quartile range of the PTT between thumbs to ear was 50–70 ms and with a median at 60 ms. It is also reported that the PTT between face area and hand area is within the range from 9.9 to 99 ms [32]. Therefore, IC-PTT values measured in this work are in a reasonable range.

### 3.4. IC-PTT vs. BP

**Figure 8:**
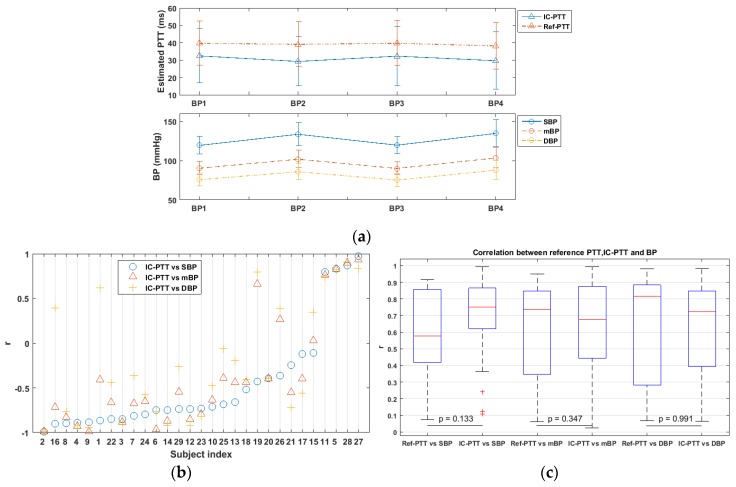
Correlation between IC-PTT (and Ref-PTT) and BP. (**a**) PTT (both IC-PTT and Ref-PTT) varied with BP (systolic BP (SBP), diastolic BP (DBP), and mean BP (mBP)) over time during the experiment; (**b**) Correlation coefficient between IC-PTT, SBP, DBP, and mBP per subject; (**c**) The absolute correlations coefficient between IC-PTT and BP (SBP, DBP, and mBP) and that between Ref-PTT and BP (SBP, DBP, and mBP).

Figure 8a shows the IC-PTT and the Ref-PTT values (mean and standard deviation) over all subjects for each BP measurement. We also found that the mean variation of SBP (21.1 ± 11.02 mmHg) was larger than that of DBP (15.47± 10.27 mmHg). A mean BP (mBP) is employed as an indicator of the general BP level, which is calculated as: mBP = 1/3 × SBP + 2/3 × DBP [48]. It shows that both IC-PTT and Ref-PTT varied inversely with BP changes, and the Ref-PTT values have a smaller variability than the IC-PTT values.

The correlation coefficients for each subject between IC-PTT and BP (i.e., SBP, DBP, and mBP) that was estimated based upon all four BP measures are shown in Figure 8b. Over all subjects, the correlation coefficient (r) between IC-PTT and SBP was −0.4549 ± 0.5871 (from −0.9934 to 0.9752), IC-PTT and DBP was −0.2258 ± 0.6524 (from −0.9829 to 0.9089), and IC-PTT and mBP was −0.3690 ± 0.6194 (from −0.9932 to 0.9297). The highest correlation appeared between IC-PTT and SBP, confirming the finding in the literature [32], where a correlation coefficient of −0.80 ± 0.12 between PTT and SBP and of −0.36 ± 0.25 between PTT and DBP were reported.

The boxplot of the correlation coefficient between BP (i.e., SBP, DBP, and mBP) and PTT measured by different systems (i.e., IC-PTT and Ref-PTT) is shown in Figure 8c. In general, a relatively strong correlation (r > 0.6) between Ref-PTT and BP and between IC-PTT and BP were observed in most subjects. Moreover, no significant differences in correlation coefficient were found between using the ICPPG system and the reference system (examined with a Wilcoxon test). This means that our proposed ICPPG system can be an alternative of the system using multiple cPPG sensors for PTT and BP measurements.

## 4. Discussion

Previous work report ventricle and arteries are common determinants of pulse arrival time (PAT, the time that ABP pulse transits from the heart to an arterial site) and SBP instead of DBP [49]. The experiment in this study indicates that exercises would not lead to marked changes in DBP so that the PTT induced by physical exercises is less correlated with DBP than SBP. Moreover, as shown in Figure 8c, IC-PTT vs. DBP correlation is the lowest compared with IC-PTT vs. SBP and IC-PTT vs. mBP, which is consistent with previous conclusions. However, the reference system shows contrary results where Ref-PTT has the highest correlation with DBP. The Willcoxon test indicates no significant difference between the correlation with DBP and the PTT derived by both systems. That could mean the Ref-PTT is suboptimal in estimating the SBP to some extent.

The previous work concludes that experimentally measured PTT generally decreases with increasing BP [49]. However, in this study, for some subjects, we found that IC-PTT (and Ref-PTT) was increased along with increasing BP, which shows a positive correlation between IC-PTT and BP. This result is not consistent with empirical conclusion from previous work. Reasons such as age and gender were excluded due to the similar age group in this study. Moreover, it seems not simply induced by measurement errors because it occurred also in the correlation between the Ref-PTT and BP. In this study, the PTT is calculated from a pair of PPG signals collected from the head (cheek or temple) and the hand area (fingertip or wrist), respectively. In other words, it is the difference between the heart-to-face transit time and the heart-to-hand transit time of the ABP pulse. The transit time that ABP pulse transits from heart to an arterial site is called the pulse arrival time (PAT), which can be measured by an electrocardiogram (ECG) signal and a PPG signal. A possible hypothesis or explanation of the positive correlation is that the PAT response to BP variation in different artery branches could differ, higher for the head branch than the hand branch, as speculated for those subjects. Consequently, with increasing (or decreasing) BP, the time that the ABP pulse travels from heart to head decreased (or increased) much more than it travels to the hand. To verify this, we employed a dataset used in our previous study, containing BP, ECG and PPG signals collected at ear and wrist from 20 subjects [50]. Through the ECG signal and PPG signals at the earlobe and the wrist, the PAT values for each arterial branch were uncovered. It was found that the PAT of the earlobe decreases faster than the wrist when BP is rising for some subjects. Additionally, we found that the PTT between the earlobe and the wrist shows a positive correlation with BP for those subjects, verifying the hypothesis we made.

In this study, some issues merit future investigation. For example, first, the reference system employed here is not the gold standard to produce the PTT. The reason we selected the contact sensor as a reference is based on the validated result of using two cPPG signals to measure PTT for BP estimation in literature [8,9,10,11,12,13,14,15,16,32]. Second, due to the limitation of our set-up, PPG signals of the hand area and head area acquired by the ICPPG system and reference system might not be exactly from same arterial sites. It might lead to errors in calculating IC-PTT and Ref-PTT values. Finally, our dataset mainly contains a young population (from 20 to 30 years of age). In future work, elderly populations should be taken into account. Last but not least, the effect of the up-sampling technique in the algorithm of ABP beats detection can be further investigated and compared with other interpolation techniques.

## 5. Conclusions

This work validated the feasibility of a novel ICPPG system using PPG signals acquired by two sensors with different modalities (i.e., imaging and contact PPGs) to estimate BP. The PPG signals were collected at two different arterial sites (the head and the hand) from 29 subjects during several alternating relaxation and exercise sessions. It is experimentally shown that the ICPPG system is able to produce a reliable PTT measurement, which coincides with the reference measures. The measured PTT values through the ICPPG system showed a significant correlation (r > 0.6) with SBP for 75.9% of the subjects, with DBP for 55.2% of the subjects, and with mBP for 65.5% of the subjects. The most important advantage of the proposed ICPPG is of unobtrusiveness in PTT and BP monitoring compared to the existing methods using multiple cPPG or multiple iPPG sensors. It is expected to open up a new path for future research in the field of continuous and wearable BP measurement.

## Appendix A

In all 29 subjects, the estimated PTT values from the reference system (Ref-PTT) and the proposed system (IC-PTT), and corresponding BP values for each blood pressure measurement were recorded as the Table A1.

**Table A1:** Supplementary data of PTT values and BP values for all participants.

Subject Index		Phase I	Phase II	Phase III	Phase IV
1	Ref-PTT (ms)	49.41	50.98	51.27	45.83
	IC-PTT (ms)	28.28	29.81	28.84	31.76
	SBP (mmHg)	124.00	125.00	122.00	109.00
	DBP (mmHg)	81.00	86.00	81.00	84.00
2	Ref-PTT (ms)	49.19	42.38	44.93	42.25
	IC-PTT (ms)	55.54	39.33	50.39	42.26
	SBP (mmHg)	119.00	141.00	124.00	135.00
	DBP (mmHg)	79.00	101.00	82.00	98.00
3	Ref-PTT (ms)	51.09	48.80	50.54	47.52
	IC-PTT (ms)	52.80	46.87	59.04	49.55
	SBP (mmHg)	112.00	147.00	114.00	141.00
	DBP (mmHg)	69.00	87.00	64.00	90.00
4	Ref-PTT (ms)	30.51	32.65	30.97	26.22
	IC-PTT (ms)	23.67	25.51	24.19	11.00
	SBP (mmHg)	117.00	119.00	119.00	123.00
	DBP (mmHg)	78.00	81.00	76.00	92.00
5	Ref-PTT (ms)	39.69	39.97	35.78	40.79
	IC-PTT (ms)	20.27	22.58	20.41	22.18
	SBP (mmHg)	124.00	141.00	124.00	128.00
	DBP (mmHg)	75.00	90.00	77.00	79.00
6	Ref-PTT (ms)	45.49	47.26	47.78	48.78
	IC-PTT (ms)	40.35	28.38	31.66	32.38
	SBP (mmHg)	111.00	123.00	116.00	126.00
	DBP (mmHg)	68.00	75.00	72.00	68.00
7	Ref-PTT (ms)	49.00	48.26	47.11	47.14
	IC-PTT (ms)	10.43	-0.25	8.33	-10.79
	SBP (mmHg)	141.00	161.00	141.00	157.00
	DBP (mmHg)	91.00	95.00	87.00	91.00
8	Ref-PTT (ms)	31.18	36.75	38.32	39.33
	IC-PTT (ms)	24.27	17.70	22.77	23.34
	SBP (mmHg)	121.00	148.00	119.00	131.00
	DBP (mmHg)	78.00	92.00	75.00	86.00
9	Ref-PTT (ms)	37.44	35.57	35.26	31.32
	IC-PTT (ms)	24.81	21.61	24.42	21.89
	SBP (mmHg)	128.00	150.00	135.00	167.00
	DBP (mmHg)	89.00	108.00	86.00	100.00
10	Ref-PTT (ms)	48.35	54.58	56.76	52.51
	IC-PTT (ms)	66.13	56.59	65.55	61.24
	SBP (mmHg)	115.00	138.00	115.00	153.00
	DBP (mmHg)	81.00	83.00	77.00	90.00
11	Ref-PTT (ms)	28.88	24.96	28.71	27.24
	IC-PTT (ms)	9.03	12.41	3.97	10.62
	SBP (mmHg)	107.00	132.00	108.00	129.00
	DBP (mmHg)	71.00	92.00	73.00	94.00
12	Ref-PTT (ms)	16.79	18.48	17.40	12.77
	IC-PTT (ms)	41.71	34.92	49.41	32.93
	SBP (mmHg)	136.00	152.00	140.00	148.00
	DBP (mmHg)	92.00	101.00	91.00	100.00
13	Ref-PTT (ms)	47.53	46.30	39.88	46.41
	IC-PTT (ms)	53.66	45.93	53.54	54.61
	SBP (mmHg)	132.00	143.00	132.00	140.00
	DBP (mmHg)	84.00	84.00	77.00	85.00
14	Ref-PTT (ms)	53.55	42.06	52.00	42.86
	IC-PTT (ms)	49.18	39.92	47.99	41.33
	SBP (mmHg)	145.00	152.00	137.00	166.00
	DBP (mmHg)	69.00	85.00	67.00	92.00
15	Ref-PTT (ms)	34.65	37.58	40.55	42.32
	IC-PTT (ms)	45.24	42.99	55.40	50.73
	SBP (mmHg)	123.00	138.00	122.00	153.00
	DBP (mmHg)	84.00	86.00	86.00	91.00
16	Ref-PTT (ms)	56.01	56.06	54.66	51.76
	IC-PTT (ms)	59.52	56.24	62.40	59.33
	SBP (mmHg)	117.00	122.00	112.00	121.00
	DBP (mmHg)	67.00	68.00	69.00	69.00
17	Ref-PTT (ms)	30.00	30.59	33.10	25.07
	IC-PTT (ms)	16.62	17.53	18.54	16.52
	SBP (mmHg)	113.00	117.00	122.00	132.00
	DBP (mmHg)	72.00	77.00	71.00	83.00
18	Ref-PTT (ms)	30.30	30.46	44.13	38.10
	IC-PTT (ms)	30.55	20.38	29.53	28.06
	SBP (mmHg)	124.00	152.00	124.00	166.00
	DBP (mmHg)	76.00	99.00	75.00	119.00
19	Ref-PTT (ms)	53.45	53.32	52.88	51.84
	IC-PTT (ms)	43.53	40.99	34.82	49.12
	SBP (mmHg)	112.00	115.00	118.00	116.00
	DBP (mmHg)	71.00	75.00	68.00	76.00
20	Ref-PTT (ms)	27.33	25.20	27.80	28.20
	IC-PTT (ms)	33.99	31.20	33.60	28.33
	SBP (mmHg)	128.00	136.00	124.00	129.00
	DBP (mmHg)	72.00	81.00	71.00	74.00
21	Ref-PTT (ms)	19.22	14.78	15.90	13.85
	IC-PTT (ms)	16.82	0.33	3.07	1.00
	SBP (mmHg)	112.00	116.00	103.00	123.00
	DBP (mmHg)	72.00	79.00	75.00	86.00
22	Ref-PTT (ms)	42.03	42.98	49.69	46.19
	IC-PTT (ms)	22.39	14.65	36.30	22.52
	SBP (mmHg)	133.00	145.00	130.00	134.00
	DBP (mmHg)	91.00	99.00	94.00	93.00
23	Ref-PTT (ms)	22.57	23.90	20.55	24.52
	IC-PTT (ms)	12.40	8.78	8.44	6.14
	SBP (mmHg)	101.00	131.00	102.00	138.00
	DBP (mmHg)	66.00	93.00	71.00	109.00
24	Ref-PTT (ms)	25.74	20.81	21.29	−1.10
	IC-PTT (ms)	13.55	14.14	13.63	12.59
	SBP (mmHg)	114.00	115.00	109.00	128.00
	DBP (mmHg)	68.00	77.00	66.00	86.00
25	Ref-PTT (ms)	50.79	49.64	52.70	49.20
	IC-PTT (ms)	36.63	31.62	22.39	22.69
	SBP (mmHg)	112.00	121.00	118.00	124.00
	DBP (mmHg)	69.00	73.00	68.00	73.00
26	Ref-PTT (ms)	53.83	52.10	54.84	51.37
	IC-PTT (ms)	30.49	27.02	26.15	31.87
	SBP (mmHg)	98.00	104.00	101.00	102.00
	DBP (mmHg)	62.00	70.00	61.00	72.00
27	Ref-PTT (ms)	31.09	32.88	33.52	30.64
	IC-PTT (ms)	30.37	37.09	26.94	34.98
	SBP (mmHg)	108.00	113.00	102.00	112.00
	DBP (mmHg)	67.00	76.00	69.00	78.00
28	Ref-PTT (ms)	46.56	46.33	45.72	48.43
	IC-PTT (ms)	28.03	29.05	26.81	29.53
	SBP (mmHg)	114.00	141.00	118.00	152.00
	DBP (mmHg)	76.00	93.00	75.00	105.00
29	Ref-PTT (ms)	70.30	68.36	60.34	66.37
	IC-PTT (ms)	37.80	23.09	28.64	34.28
	SBP (mmHg)	121.00	134.00	119.00	120.00
	DBP (mmHg)	78.00	83.00	75.00	82.00
